# PHYSICAL ACTIVITY AS A MEDIATOR BETWEEN RACE/ETHNICITY AND CHANGES IN COGNITION

**DOI:** 10.1093/geroni/igad104.3111

**Published:** 2023-12-21

**Authors:** Jason Newsom, Mallory Kroeck, Em Trubits, Jennifer Saucedo, Anda Botoseneanu, Heather Allore, Corey Nagel, Ana Quiñones

**Affiliations:** Portland State University, Portland, Oregon, United States; Portland State University, Portland, Oregon, United States; Portland State University, Portland, Oregon, United States; Portland State University, Portland, Oregon, United States; University of Michigan, Ann Arbor, Michigan, United States; Yale University, New Haven, Connecticut, United States; University of Arkansas for Medical Sciences, Little Rock, Arkansas, United States; Oregon Health & Science University, Portland, Oregon, United States

## Abstract

Racial/ethnic disparities in the rate of cognitive decline in older age have been established. Studies also report differences in physical activity across racial/ethnic groups. We investigated whether racial/ethnic differences in changes in cognition over a 12-year period (2006-2018) were mediated by physical activity using data from the Health and Retirement Study (N = 16,777, mean baseline age = 66.0 years). Structural equation modeling was used to estimate a latent growth curve model of changes in cognitive scores (27-item TICS) and investigate whether the relationship of race/ethnicity (non-Hispanic Black, Hispanic, non-Hispanic White) to change in cognition was mediated by physical activity after controlling for age, sex, education, marital status, personal wealth, and insurance coverage. Results indicated that Blacks engaged in significantly lower levels of physical activity than Whites (b = -.176, ♌ = -.123, p < .001), but there were no differences between Hispanics and Whites (b = .028, ♌ = .017, ns). Physical activity significantly predicted higher initial cognitive scores (b = 5.205, ♌ = .610, p < .001) and less decline in cognitive scores over time (b = 1.063, ♌ = .796, p < .001). The indirect (mediational) effect for the Black vs. White comparison on changes in cognitive score was significant (b = .187, ♌ = .152, 95% CI [.135,.244]). These results provide important new information for understanding how physical activity, a modifiable lifestyle factor, may help explain racial/ethnic disparities in cognitive decline in middle and later life, suggesting greater need to reduce sedentary behavior and increase activity.

